# Latent profiles of mental health and care barriers among heterosexual and sexual minority adolescents

**DOI:** 10.1186/s13584-026-00781-0

**Published:** 2026-09-04

**Authors:** Ateret Gewirtz-Meydan, Ruth Berkowitz, Shir Maoz, Guy Shilo, Yehuda Neumark

**Affiliations:** 1 School of Social Work, Faculty of Social Welfare and Health Sciences, University of Haifa, Haifa, Israel; 2 School of Social Work, Faculty of Social Sciences, Tel-Aviv University, Tel- Aviv, Israel; 3 Braun School of Public Health and Community Medicine, Hebrew University of Jerusalem, Jerusalem, Israel

**Keywords:** Adolescents, Mental health help-seeking, Sexual minority youth, Latent profile analysis, Barriers to care

## Abstract

**Background:**

Adolescents often navigate complex psychological, behavioral, and identity-related challenges, yet many do not seek the mental health support they need. Sexual minority youth face additional identity-based barriers that further hinder access to care.

**Method:**

A sample of 1,129 Israeli adolescents aged 16–18 (M = 17.42, SD = 1.03), including 30% who identified as sexual minorities, completed measures of mental health, substance use, suicidal ideation, and perceived barriers to care. Latent Profile Analysis (LPA) identified subgroups based on mental health and behavioral risk, and Exploratory Graph Analysis (EGA) identified latent dimensions of help-seeking barriers. Moderation analyses tested whether sexual orientation shaped associations between profile membership, perceived barriers, and help-seeking intentions.

**Results:**

Four mental health profiles emerged: Within Normal Ranges, Emotional Struggles and Suicidal Ideation, Tobacco and E-cigarette Users, and Cannabis Users. Barriers to care clustered into three domains: Identity-Related Barriers, Accessibility and Structural Barriers, and Emotional and Internal Barriers. Higher-risk profiles reported greater barriers and lower intentions to seek help, with stronger effects among sexual minority youth. Sexual orientation moderated these associations, revealing lower help-seeking intentions among sexual minority Tobacco and E-cigarette Users but higher intentions among sexual minority Cannabis Users.

**Conclusion:**

Patterns of psychological distress, substance use, and marginalized identity intersect to shape adolescents’ access to mental health care. Tailored, identity-affirming outreach and efforts to reduce structural and emotional barriers are essential for reaching youth who face the greatest obstacles to seeking support.

**Supplementary Information:**

The online version contains supplementary material available at https://doi.org/10.1186/s13584-026-00781-0.

## Public policy implications

This study maps four patterns of adolescent mental health risk and three kinds of barriers to care, showing that LGBTQ+ youth in higher-risk groups face stronger identity-related and emotional obstacles and are less likely to seek help. Israel’s Ministry of Health, health funds, Ministry of Education, and local service providers should coordinate multidomain screening and referral pathways that consider the different barriers preventing adolescents from engaging with care. Schools and community services should provide confidential, low-threshold access, while professional training standards should require LGBTQ-affirming and identity-safe care. Following prospective validation, profile-informed assessment could be piloted in Israel and adapted by other national health systems.

## Introduction

Adolescence represents a critical developmental window in which patterns of health behavior, identity formation, and psychological vulnerability often intersect in complex ways. This period is characterized by increased exposure to emotional distress, behavioral dysregulation, and risk-taking behaviors, including substance use and suicidal ideation [72]. Association with antisocial peers, absence of positive role models, and strained parent–child relationships can serve as significant risk factors for substance use [12]. Although early identification and intervention can significantly mitigate long-term psychological harm [8, 40], adolescents frequently underutilize mental health services, despite high levels of need [59]. Barriers to accessing care range from practical challenges, such as transportation and affordability, to more nuanced psychological and sociocultural factors, including fear of judgment, lack of perceived relevance, and identity-based concerns [44].

For sexual minority youth, these barriers are intensified by minority stressors, including internalized stigma, anticipated rejection, and institutional invisibility [57, 62]. These barriers and stressors compound existing risks and have been linked to elevated rates of mental health problems [36, 54, 61], including increased rates of suicidality and higher incidence of suicide attempts [31, 32, 35, 39, 42, 55, 67], and substance use [5, 51, 63]. Moreover, sexual minority adolescents often report identity-related fears, such as not being understood or accepted by mental health professionals, as central to their reluctance to seek support [7, 14, 28, 41, 44, 69]. Yet, the interaction between these identity-driven concerns and comprehensive mental health profiles capture diverse subgroups of adolescents with distinct patterns of symptoms and barriers to care remains underexplored.

To address this gap, the current study adopts a person-centered, latent profile analysis (LPA) approach to identify meaningful subgroups of adolescents based on their substance use (tobacco, alcohol, cannabis, electronic cigarette), emotional and behavioral symptoms, and suicidal ideation. Unlike traditional variable-centered methods, LPA allows for the identification of subpopulations with distinct risk constellations, which may differentially relate to internal experiences and external barriers [9, 49]. Recognizing that help-seeking is shaped not only by external accessibility but also by internalized beliefs and identity-related concerns, we further examine barriers to care using Exploratory Graph Analysis (EGA; [19]), a cutting-edge method in network psychometrics that elucidates the underlying structure of multivariate data. By applying EGA to a comprehensive list of help-seeking barriers, we seek to identify latent dimensions that may capture clusters of concern, thereby offering a nuanced view of the psychosocial ecology in which mental health decisions are made [6]. Importantly, we also test whether sexual orientation moderates the association between risk profile membership and (1) perceived barriers to care, and (2) intention to seek mental health help.

## The study context

This study was conducted in Israel, a culturally and religiously diverse society that includes Jewish, Muslim, Christian, and Druze communities. Although legislation supporting LGBTQ+ rights has advanced in recent years, societal acceptance remains limited, and sexual minority adolescents often experience verbal harassment and emotional insecurity (The Aguda – The Association for LGBTQ Equality in Israel, [64]). These challenges intersect with broader adolescent risk behaviors. Recent findings from the Israeli Health Behaviour in School-aged Children (HBSC) survey, conducted in 2023, indicate that approximately 13% of adolescents reported binge drinking during the previous 30 days, 8.4% reported lifetime cannabis use (6% during the previous month), and experimentation with cigarettes and e-cigarettes was reported by approximately 14% and 16% of adolescents, respectively (Health Behaviour in School-aged Children Israel, [26]. Consistent with these findings, a study conducted during the COVID-19 pandemic found that 31% of Israeli adolescents reported psychoactive substance use involving alcohol, tobacco, cannabis, or cocaine [13]. In parallel, daily mental distress among adolescents increased sharply, from 20% in 2019 to approximately 30% in 2022, alongside rising rates of nervousness, low mood, and sleep difficulties (World Health Organization Regional Office for Europe, [73]. This complex landscape underscores how co-occurring risk behaviors and mental health challenges may intensify minority stress and further restrict access to support for sexual minority adolescents [43]. Although research increasingly uses person-centered approaches to identify meaningful profiles and subgroups of adolescents, clinical assessment and referral still tend to focus on individual symptoms or immediate risk. The present study contributes to the evidence base needed to support more differentiated and tailored referral practices, although further validation is required before such profiles can be applied clinically.

### Method

### Participants

The study sample consisted of 1,129 Israeli adolescents aged 16–18 (M = 17.42, SD = 1.03). To better understand help-seeking among sexual minority youth, we intentionally oversampled this group thorough social media promotion. 338 participants (30%) identified as sexual minority, including gay, bisexual, queer, pansexual, questioning, or other orientations, while 70% identified as heterosexual.

This proportion is substantially higher than available estimates. While Israel lacks a national system for systematically tracking sexual minority identification, recent estimates from the U.K. and U.S. suggest that between 15.2% and 17% of young adults identify as LGBTQ+ [15, 33]. Additionally, a 2025 report by the Williams Institute found that 3.3% of youth aged 13 to 17 in the United States identify as transgender [27]. Thus, the data are not intended to be representative of the general Israeli population aged 16–18, but rather to provide meaningful insights into the experiences of sexual minority youth and the barriers they face. Table 1 provides a comprehensive breakdown of demographic characteristics of the study participants.

### Measures

*Sociodemographic Characteristics* included age, sex assigned at birth, gender identity, sexual orientation, religion, religiosity, and parental marital status. Sexual orientation was assessed using an inclusive question modeled after previous research [11, 68]. “People describe their sexual orientation in different ways. Which expression best describes your current sexual orientation?” Response options included heterosexual/straight, gay or lesbian, mainly heterosexual, mainly gay or lesbian, bisexual, queer, pansexual, polysexual, asexual, and “I do not know yet or I am currently questioning my sexual orientation.” Participants could also select “None of the above” and describe their orientation in an open-text field or indicate that they preferred not to answer. The sociodemographic characteristics of the sample are presented in Table 1.

*Mental Health Difficulties* were assessed using the Strengths and Difficulties Questionnaire (SDQ; [20]), a highly acclaimed and widely utilized instrument for population-based mental health surveys. The SDQ is a 25-item self-report questionnaire comprising five subscales that assess difficulties in four psychological domains: emotional symptoms (e.g., “I have many fears, I am easily scared”; “I get a lot of headaches, stomach-aches or sickness”), hyperactivity/inattention (e.g., “I am restless, I cannot stay still for long”; “I finish the work I’m doing. My attention is good”), conduct problems (e.g., “I take things that are not mine from home, school or elsewhere”; “I get very angry and often lose my temper”), and peer problems (e.g., “I am rather solitary, I tend to play alone”; “Other people my age generally like me”), as well as strengths in prosocial behavior (e.g., “I try to be nice to other people. I care about their feelings”; “I am helpful if someone is hurt, upset or feeling ill”). Respondents were asked to rate their experiences over the past six months, using a 3-point Likert scale (0 = not true, 1 = somewhat true, 2 = certainly true). The Hebrew version of the SDQ was validated in a population-based study of adolescents and their mothers, demonstrating good internal consistency with a Cronbach’s alpha coefficient of 0.77 for the total difficulties scale [37]. In the current study, internal consistency for the SDQ subscales ranged from poor to acceptable, with emotional symptoms (α = 0.71), conduct problems (α = 0.51), hyperactivity/inattention (α = 0.69), peer problems (α = 0.57), and prosocial behavior (α = 0.63).

*Suicidal Ideation* was assessed using the Ask Suicide-Screening Questions (ASQ) ([29]; National Institute of Mental Health; [65], a 4-item suicide-risk screening instrument. The ASQ items inquire about wishes to be dead, feelings of oneself or others being better off if one was dead, thoughts of killing oneself and previous attempts at killing oneself. If a patient’s response is “no” to all 4 items, this is considered a negative screen. If a patient responds “yes” to any item or refuses to answer, the screen is considered “positive”. A fifth question, which is used to determine acuity in cases of a positive screen (“Are you having thoughts of killing yourself right now?”), was removed from the current study due to ethical concerns. The tool has been validated for use in inpatient and outpatient settings for ages 10 to 21, with sensitivity values ≥ 95% and specificity values ≥ 87% [2]. In our current study, internal consistency of the ASQ scale was acceptable (α = 0.70).

*Substance Use* was assessed using a self-report questionnaire, focusing on the frequency and types of substance use over the past three months. Participants were asked to indicate how often they used various substances, including tobacco products (such as cigarettes, hookah, cigars), alcoholic beverages (including beer, wine, spirits, or mixed drinks), illegal drugs or substances (such as marijuana, hashish, ecstasy, or other designer drugs), and electronic cigarettes. Responses were rated on a 5-point frequency scale (1 = not at all, 2 = once or twice in the past three months, 3 = every month, 4 = every week, 5 = every day or almost every day). Thus, the substance-use indicators captured frequency of use during the previous three months but did not assess quantity per occasion, motives for use, social context, or substance-related impairment. An open-text field was included to allow participants to report any other substances they had used that were not listed in the questionnaire. For each participant, an individual substance use score was calculated by averaging their responses across all substance use items. Internal consistency for this section was found to be acceptable (α = 0.89).

*Barriers to Accessing Mental Health Care* were assessed using a 26-item scale developed and validated specifically for the current study [4]. The scale was designed to capture a comprehensive range of barriers that youth face when seeking mental health support. Items were informed by prior research on general barriers to care [69], which include not knowing whom to contact, lack of transportation, unavailability of someone to accompany them, parental refusal, a desire to keep parents unaware, difficulty making appointments, fear of a provider’s response or actions, belief that the problem will resolve on its own, inability to afford services, and other obstacles. In addition, we incorporated barriers identified by LGBTQ+ youth based on insights from The Trevor Project [47], such as fear of being outed and concerns about identity misunderstanding. These additions ensured that the measure captures the unique challenges faced by sexual minority youth. To strengthen the content validity of the scale, we consulted five experienced professionals working with LGBTQ+ adolescents, including psychotherapists, a university-based researcher, two clinical psychologists, and a teacher involved with at-risk youth and an active member of Hoshen - an Israeli LGBTQ education organization. In addition, we sought feedback from several LGBTQ+ adults and five adolescents (three boys and two girls, aged 16–18, corresponding to grades 10–12), with an emphasis on diversity in gender and age to reflect the target population.

Each item was rated on a 5-point Likert scale (1 = strongly disagree, 2 = disagree, 3 = neutral, 4 = agree, 5 = strongly agree), with higher scores indicating greater perceived barriers. To examine these barriers across profiles, we conducted an EGA (see data analysis section below for details). The analysis yielded a three-factor solution: (1) accessibility and structural barriers (8 items; e.g., *“My parents or guardian cannot afford such service”*; α = 0.90), (2) emotional and internal barriers (12 items; e.g., *“I’m afraid my friends or relatives will find out”*; α = 0.76), and (3) identity-related barriers (6 items; e.g., *“I feel the professional won’t understand my identity”*; α = 0.76).

*Intentions to Seek Mental Health Help* was assessed using three items adapted from the Mental Help-Seeking Intention Scale (MHSIS; [24]). These items measured the likelihood of seeking professional help when experiencing psychological difficulties. Participants rated the statements, “If I were experiencing psychological difficulties, I would consider seeking help from a mental health professional” and “If I were experiencing psychological difficulties, I would try to seek help from a mental health professional,” on a 7-point Likert scale (1 = strongly disagree, 2 = disagree, 3 = somewhat disagree, 4 = neutral, 5 = somewhat agree, 6 = agree, 7 = strongly agree). Together, the items reflect both openness to and inclination toward seeking help, with higher scores indicated stronger intentions to seek professional mental health support. The correlation of the two items was high (*r* = .96).

### Procedure

Recruitment of youth participants was conducted through paid advertisements managed via META’s advertising platforms and delivered on Instagram. Instagram was selected because it is among the most widely used social media platforms among Israeli adolescents (Israel Internet Association, [30]), consistent with international evidence on adolescents’ social media use (Pew Research Center, [52]). This approach ensured broad reach and relevance for the target demographic. Clicking on an ad directed potential participants to a secure survey website hosted on the Qualtrics platform, where they completed a short screener survey. Eligible youth, defined as those aged 16 to 18, were presented with an informed consent form and invited to participate. For inclusivity, the survey was available in both Hebrew and Arabic. Those who provided electronic consent were directed to the main behavioral survey. Participants were allowed to pause and return to complete the survey at their convenience. Incentives were offered in the form of a raffle for twenty 50 NIS gift cards upon survey completion. To preserve anonymity, participants who wished to enter the raffle were directed, upon completing the main survey, to a separate form in which they could voluntarily provide their contact information; this information was not linked to their survey responses. The survey took an average of 15 min to complete.

Ethical considerations included a waiver of parental consent for youth participation, approved by the ethics committee of the Faculty of Social Welfare and Health Sciences [Approval number: 404/23]. This waiver was granted due to the potential risks of forced disclosure and harm associated with requiring parental permission, particularly for sexual minority youth [11, 75]. Prior research has shown that requiring parental consent can result in significant nonparticipation among closeted youth, introducing sampling bias [16].

### Data analysis

The analyses were conducted with 1,129 participants. Before the primary analyses, we examined the normal distribution of all main study measures using a series of Anderson-Darling normality tests. We also assessed the presence of multivariate outliers using the Minimum Covariance Determinant approach (performed with the *MASS* R package). We found that all measures significantly deviated from normality (all *A* > 2.76, all *p* < 5.80^^−7^ or lower). In addition, 180 observations were identified as multivariate outliers. Accordingly, we used robust statistics to examine the study’s hypotheses. Additionally, missing data were permitted, resulting in 4.9% of the data being absent, with 25 distinct patterns. Jamshidian and Jalal’s non-parametric Missing Completely at Random (MCAR) test indicated that the data were missing completely at random (*Hawkins’s χ*^*2*^_(10)median_ = 43.70, *p*_median_ = 3.72^^−6^, *Anderson-Darling T*_median_ = 4.86, *p*_median_ = 0.236). Therefore, we addressed missing data using the Multiple Imputation procedure [60] with five complete sets in keeping with the percentage of missing data, which were implemented using the *mice* and *micemd* R packages. The reported results represent the pooled outcome of the MI procedure.

**Fig. 1:**
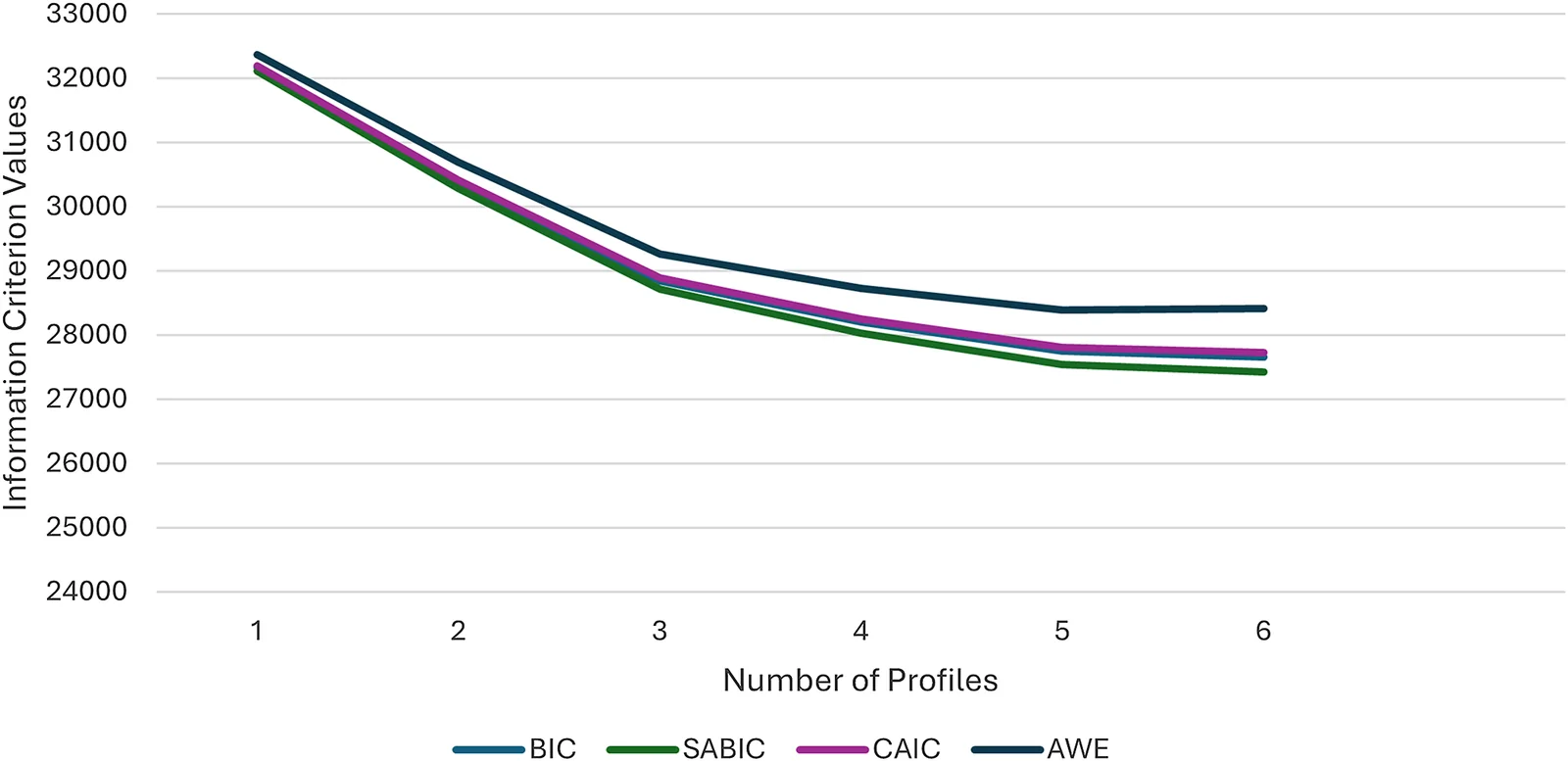
Information criteria scree plot

We began by applying latent profile analysis to estimate distinct latent profiles in participants’ self-reported substance use (tobacco, alcohol, cannabis, and electronic cigarette), mental health (emotional symptoms, conduct problems, hyperactivity/inattention, peer relationship problems, and prosocial behavior), and suicidal ideation (yes, no). To do this, we follow the guidelines of Nylund-Gibson and Choi [49] using the *tidyLPA* R package with MPlus 8.8 Structural Equation Modeling (SEM) integration [45]. We examined one to six possible profiles using unconditional LPA. To decide on the number of profiles, we used the following information (summarized in Table 2): (i) information criteria (IC) – including the Bayesian Information Criterion (BIC), Sample-size adjusted Bayesian Information Criterion (SABIC), Consistent Akaike Information Criterion (CAIC), and Approximate Weight of Evidence Criterion (AWE) – which are approximate fit indices where lower values indicate superior fit. These IC were also plotted (see Fig. 1) to inspect for an “elbow” of point of “diminishing returns” in model fit (equivalent to a scree plot in factor analysis). (ii) We also used the bootstrapped likelihood ratio test (BLRT), which provides *p*-values to assess whether adding a class leads to a statistically significant improvement in model fit. The BLRT is one of the most robust methods across various modeling conditions [48]. (iii) We considered the Bayes Factor (BF) indices for pairwise comparisons of fit between two adjacent class models, with values greater than 10 suggesting “strong” support for the more complex model. Additionally, we assessed the correct model probability (cmP), which estimates the likelihood of each model being “correct” among all models evaluated. We also considered how the selected models relate to each other (e.g., theoretically different) as well as the relative sizes of the emergent classes. Here, we established a minimum profile size of 56 participants, representing 5% of the sample.

After identifying the optimal number of profiles, we conducted an EGA [19] using the *EGAnet* R package to determine the ideal number of factors among the 26 items evaluating participants’ barriers to seeking health care. EGA is a method in network psychometrics that employs undirected network models to assess the psychometric properties of questionnaires [18]. EGA was utilized to verify the number of factors or components using the graphical LASSO [17] and the items associated with each factor. Network loadings are roughly equivalent to factor loadings, with suggested general effect size guidelines for network loadings of 0.15 for small, 0.25 for moderate, and 0.35 for large [6]. We used the EGA results to calculate scores for each participant reflecting the latent factors underlying the barriers to seeking health care.

In the next phase, we examined differences between profiles in three areas: (i) the latent factors underlying barriers to seeking health care, (ii) participants’ intentions to seek mental health help, and (iii) sexual orientation. For analysis purposes and to allow meaningful comparisons, participants were categorized into two groups: heterosexual youth and sexual minority youth (which combined all other sexual orientations).

To assess differences in the quantitative variables, we conducted a series of one-way analyses of variance on trimmed means under the assumption of homoscedasticity, followed by Yuen’s test for trimmed means with Holm correction for post-hoc comparisons. For the categorical variable (sexual orientation), we performed a chi-square test of independence using a Monte Carlo simulation with 10,000 replications to estimate significance, followed by post-hoc pairwise comparisons of proportions. All p-values were adjusted using a false discovery rate (FDR) of 5% to account for multiple comparisons.

In the final part of the results, we tested whether participants’ sexual orientation moderated the association between profile membership and two outcomes: (1) the latent factors underlying barriers to seeking health care and (2) participants’ intentions to seek mental health help. To this end, we conducted a series of robust regression analyses using the *robustbase* R package, modeling the interaction between profile membership and sexual orientation as predictors of each outcome. All models controlled for sex (female vs. male). Where significant interactions emerged, we conducted simple slopes analyses using the *interactions* R package to probe the nature of the effects.

## Results

### Latent profile analysis

**Fig. 2:**
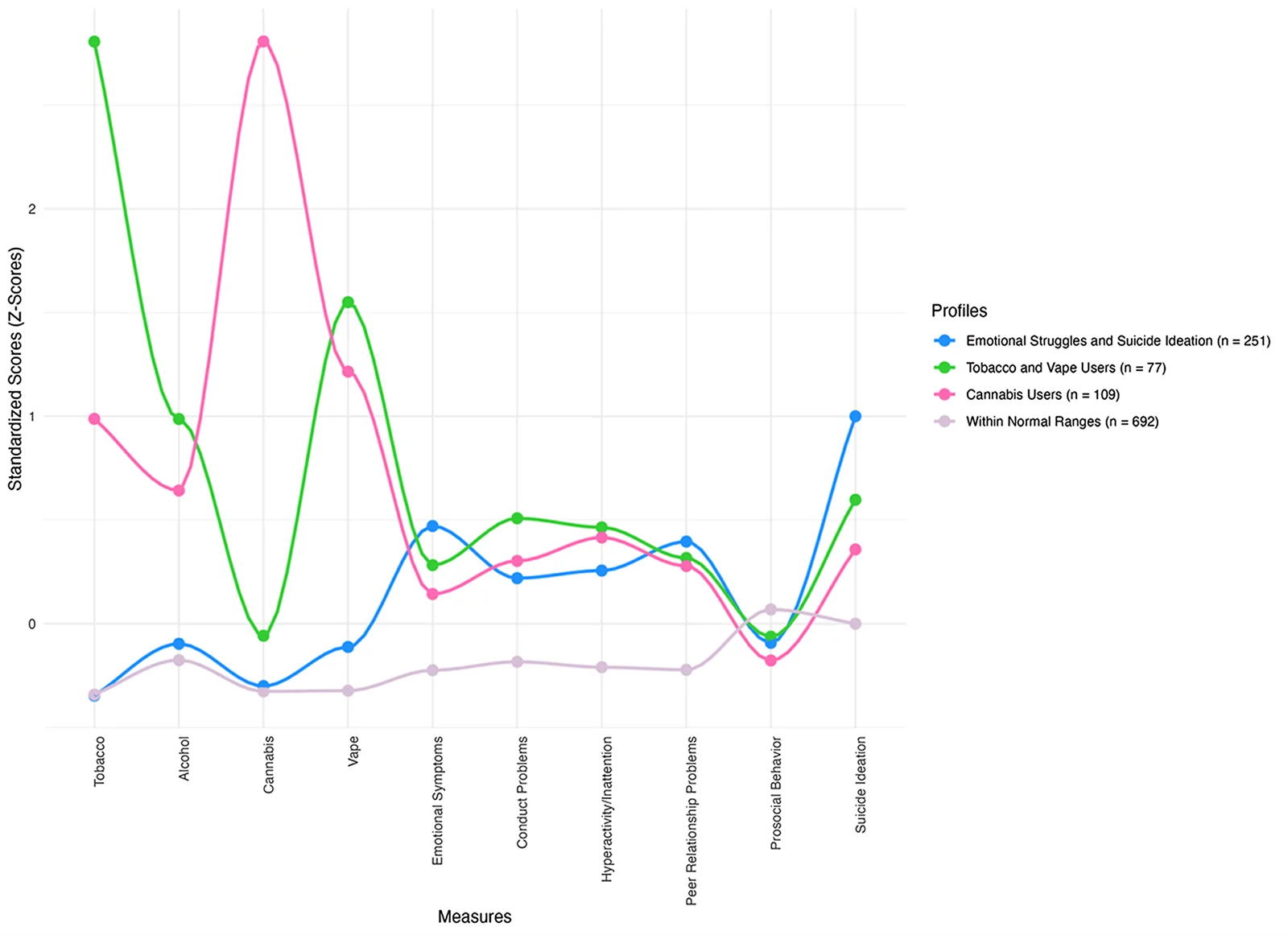
The selected 4-profile solution. The measures used in the LPA are presented on the x-axis. Relative scores are presented on the y-axis

LPA results are summarized in Table 2. As is common in applied LPA [49], the model-selection indices did not converge on a single solution. The BIC, SABIC, CAIC, and cmP favored six profiles, whereas the AWE favored five, and the BLRT supported adding a fifth profile. However, the scree plot (Fig. 1) showed an elbow at four profiles, the six-profile solution had lower entropy, and the five-profile solution contained two profiles with highly similar response patterns. Balancing statistical fit, classification quality, parsimony, profile size, and theoretical interpretability, we selected the four-profile solution, which yielded clearly differentiated profiles, adequate profile sizes, and excellent classification accuracy (entropy = 0.99; [46]). The profiles are presented in Fig. 2 and Supplementary Tables 1 and include the following groups: Within Normal Ranges (WNR; *n* = 692; 61.29%), Emotional Struggles and Suicidal Ideation (*n* = 251; 22.23%), Tobacco and E-cigarette Users (*n* = 77; 6.82%), and Cannabis Users (*n* = 109; 9.65%).

Participants in the Within Normal Ranges profile exhibited no suicidal ideation, minimal substance use, and generally favorable mental health indicators. In contrast, all youth in the Emotional Struggles and Suicidal Ideation profile reported suicidal ideation, along with the highest levels of emotional distress and peer relationship difficulties. Notably, their engagement in substance use was comparable to that of participants in the Within Normal Ranges group.

The remaining two profiles were characterized by distinct patterns of substance use accompanied by elevated conduct problems and symptoms of hyperactivity/inattention. Tobacco and E-cigarette Users reported frequent use of tobacco, vaping products, and alcohol, but low cannabis use. Conversely, Cannabis Users reported frequent cannabis and alcohol use, with minimal use of tobacco and vaping products.

## Clusters of barriers seeking health care

**Fig. 3:**
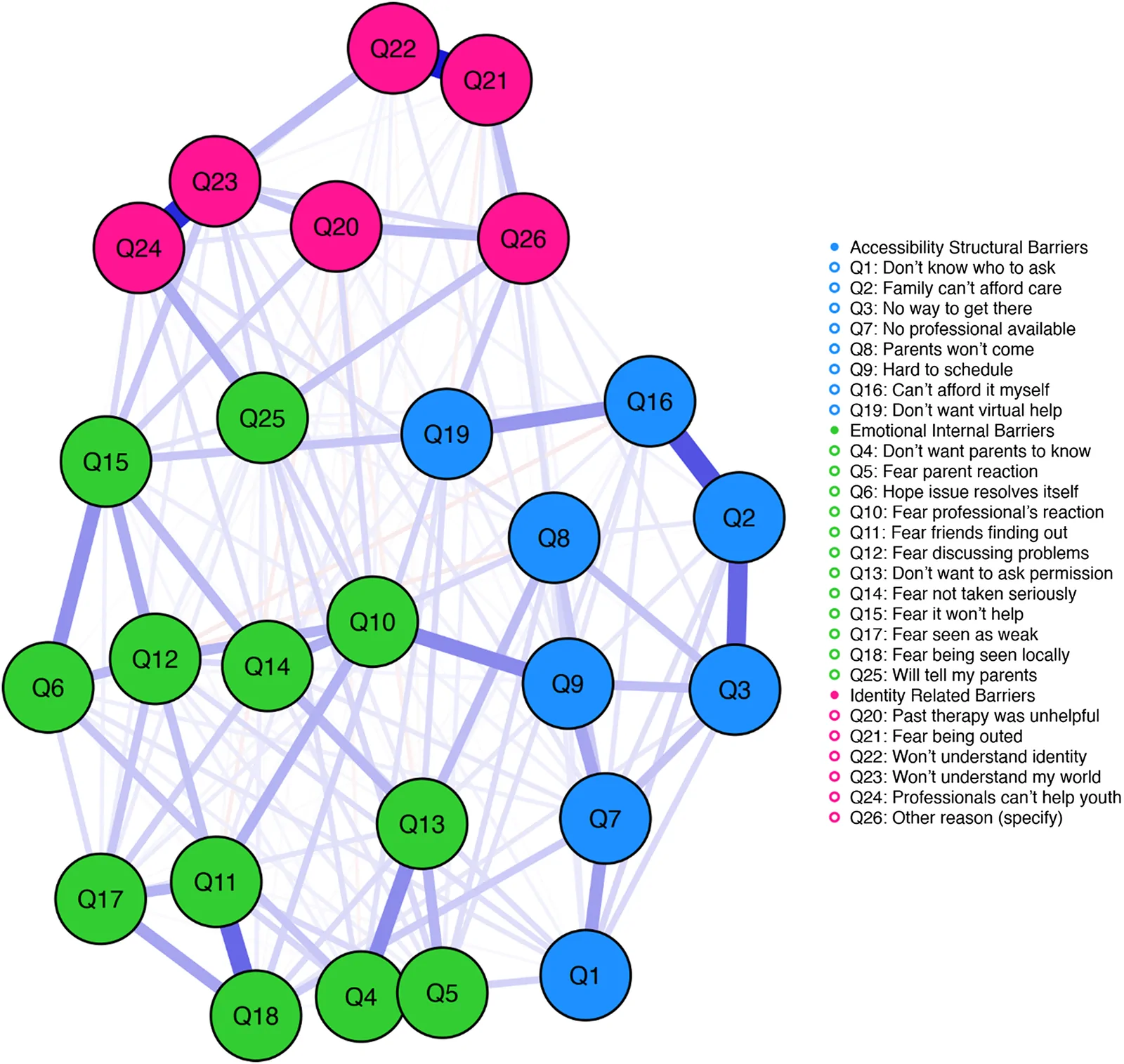
EGA results of participants’ barriers to seeking health care. Three latent factors were detected. Blue, Accessibility Structural Barriers; Green, Emotional Internal Barriers; and Pink, Identity-Related Barriers. Blue lines represent positive associations, whereas red lines represent negative associations. The length, width, and color intensity of the lines reflect the strength of the associations

The Exploratory Graph Analysis identified three latent clusters of barriers (see Fig. 3): Identity-Related Barriers (comprising six items), Accessibility and Structural Barriers (eight items), and Emotional and Internal Barriers (twelve items). The standard error for the number of dimensions was 0.78, with a 95% confidence interval ranging from 1.46 to 4.53 (median = 3). Notably, 80.42% of the iterations supported a three-factor solution.

Within the Identity-Related Barriers cluster, the items with the highest loadings were item 23 (“I don’t think the professional can understand my world; they won’t know what to say or advise”) and item 22 (“I feel the professional won’t understand my sexual orientation or gender identity”). For the Accessibility and Structural Barriers cluster, the strongest loadings were observed for item 2 (“My parents or guardian [the person responsible for me] cannot afford such a service”) and item 3 (“I have no way to get to this type of service [in terms of transportation or distance from home]”). Finally, in the Emotional and Internal Barriers cluster, the highest loadings were found for item 4 (“I don’t want my parents or guardian to know”) and item 11 (“I’m afraid my friends or relatives will find out”).

### Differences between profiles in participants’ barriers to seeking health care, intentions to seek mental health help, and sexual orientation

As shown in Table 3, significant differences emerged across profiles in all barrier dimensions, intentions to seek mental health help, and sexual orientation. These differences remained significant after applying a 5% False Discovery Rate (FDR) correction. Figure 4a and 4b illustrate the observed patterns. Participants in the Within Normal Ranges group reported the lowest levels of identity-related and accessibility structural barriers. Their emotional internal barriers were also lower than those reported by youth in the Emotional Struggles and Suicidal Ideation and Cannabis Users profiles, but not significantly different from those in the Tobacco and E-cigarette Users profile. Likewise, intentions to seek mental health help were significantly higher in the Within Normal Ranges group compared to the Emotional Struggles and Suicidal Ideation and Cannabis Users groups, but did not differ significantly from the Tobacco and E-cigarette Users group. Finally, analysis of participants’ sexual orientation revealed that the Emotional Struggles and Suicidal Ideation and Cannabis Users profiles included a higher proportion of sexual minority youth compared to the Within Normal Ranges and Tobacco and E-cigarette Users profiles.

### Differences between profiles in participants’ barriers to seeking health care and intentions to seek mental health help as a function of sexual orientation

Results are presented in Table 4. The analyses revealed significant main effects for sexual orientation on participants’ identity-related and emotional internal barriers, but not on accessibility structural barriers or intention to seek mental health help. Specifically, sexual minority youth reported greater identity-related and emotional internal barriers than heterosexuals.

These main effects, including the one on youth intentions to seek mental health help, were moderated by profile membership. Results of the simple slopes analyses are presented in Fig. 5a and 5c. We found that the difference between sexual minority and heterosexual youth was significantly greater within the Tobacco and E-cigarette Users profile than within the Within Normal Ranges profile. Specifically, among Tobacco and E-cigarette Users, sexual minority youth reported substantially higher identity-related barriers (*b* = 0.86 vs. *b* = 0.37) and emotional internal barriers (*b* = 0.19 vs. *b* = 0.12), as well as lower intentions to seek mental health help (*b* = -1.05 vs. *b* = − 0.01), compared to heterosexual youth, indicating a stronger effect of sexual orientation in this group.

The analyses also revealed that the difference between sexual minority and heterosexual youth was significantly greater within the Emotional Struggles and Suicidal Ideation profile than within the Within Normal Ranges profile in identity-related barriers. Specifically, among youth with Emotional Struggles and Suicidal Ideation, sexual minority youth reported substantially higher identity-related barriers compared to heterosexual youth (*b* = 0.63 vs. *b* = 0.37).

Finally, the analyses revealed that the difference between sexual minority and heterosexual youth was significantly greater within the Cannabis Users profile than within the Within Normal Ranges profile in youth intentions to seek mental health help. Surprisingly, among Cannabis Users, sexual minority youth reported substantially higher intentions to seek mental health help compared to heterosexual youth (*b* = 1.03 vs. *b* = − 0.01).

## Discussion

This study advances research on adolescent mental health help-seeking by integrating person-centered, network-based, and moderation analyses within a single framework. Combining LPA and EGA enabled us to identify distinct profiles of mental health, substance use, and suicidal ideation alongside clusters of barriers to care, while moderation analyses demonstrated how the associations between profile membership, barriers, and help-seeking intentions varied by sexual orientation. This integrated approach extends previous research by showing that adolescents’ barriers to care and help-seeking intentions reflect the intersection of their mental health and behavioral risk patterns with sexual-minority status. Four profiles emerged: Within Normal Ranges, Emotional Struggles and Suicidal Ideation, Tobacco and E-cigarette Users, and Cannabis Users.

The Within Normal Ranges group, representing over 60% of the sample, reported low emotional distress, minimal substance use, and the fewest barriers to accessing mental health care. This aligns onsistent with previous evidence linking relatively positive adolescent functioning with fewer perceived barriers to mental health care [21]. In contrast, youth in the Emotional Struggles and Suicidal Ideation profile demonstrated high levels of internalizing symptoms and peer difficulties, as well as elevated identity-related and emotional internal barriers. Notably, their substance-use patterns were similar to those of the Within Normal Ranges group, suggesting that elevated emotional distress and suicidal ideation do not necessarily co-occur with elevated substance use. The profiles centered around substance use (Tobacco and E-cigarette Users and Cannabis Users), presented a different pattern. These youth exhibited externalizing behaviors and attention difficulties but relatively lower levels of suicidal ideation [23, 25]. These profiles also reported elevated accessibility and structural barriers. One possible explanation is that these adolescents encounter practical obstacles to care or perceive existing services as insufficiently responsive to their needs; however, these possibilities were not directly examined.

These findings correspond with established distinctions between internalizing and externalizing pathways to psychological distress. While suicidal ideation is typically associated with internalized emotional suffering, such as hopelessness [58, 70], self-criticism [50, 74], and emotional dysregulation [10, 71], substance use is often conceptualized as an externalized coping strategy, emerging through behavioral disinhibition, and impulsivity [34, 66]. The divergence across profiles may be consistent with different patterns through which psychological and behavioral difficulties co-occur, although the cross-sectional data do not establish the mechanisms underlying these patterns.

The EGA revealed three conceptually coherent clusters of barriers: Identity-Related Barriers, Accessibility and Structural Barriers, and Emotional and Internal Barriers. These findings offer a useful taxonomy for future intervention development. Identity-related concerns, such as fears of being misunderstood due to one’s sexual or gender identity, were particularly salient among sexual minority youth, echoing prior findings [7, 14, 28]. However, our moderation analyses extend this literature by showing that the effect of sexual minority identity on help-seeking is not uniform across profiles. Specifically, Identity-Related Barriers and Emotional and Internal Barriers were significantly more pronounced among sexual minority youth in the Tobacco and E-cigarette Users and Emotional Struggles and Suicidal Ideation profiles, compared to heterosexual youth in the same groups. One possible interpretation is that these adolescents experience overlapping challenges associated with behavioral or emotional difficulties and sexual-minority status. However, experiences of marginalization were not directly assessed and should be examined in future research. This resonates with minority stress theory [43], which posits that stigma-related stress compounds general distress and contributes to avoidant health behaviors [22, 51, 53, 56].

Interestingly, among Cannabis Users, sexual minority youth reported higher help-seeking intentions than their heterosexual peers. The mechanisms underlying this unexpected finding cannot be determined from the present data. One possible explanation is greater awareness of mental health resources among some sexual-minority youth [1]; another is that cannabis use may occur alongside unmeasured needs that increase perceived need for support Benz et al., [3]. These explanations remain hypothetical and should be examined through qualitative and longitudinal research.

### Limitations and future research directions

Several limitations should be acknowledged. The cross-sectional design precludes causal inference, highlighting the need for longitudinal studies examining how mental health profiles, perceived barriers, and help-seeking intentions evolve over time. The non-representative sample also limits generalizability. Most participants identified as Jewish, participants assigned female at birth were overrepresented, and the small number of gender-minority participants prevented separate analyses. Future studies should use broader, multimethod recruitment strategies to obtain larger and more diverse samples that permit adequately powered comparisons across specific identity groups.

Combining diverse non-heterosexual identities into a single sexual-minority category may have obscured meaningful differences between groups. Furthermore, although we used one of the most comprehensive sets of response options available in the current literature [11, 68], the language used to describe sexual-minority identities continues to evolve. Indeed, a relatively large proportion of participants selected “Other” despite the extensive range of response options provided, suggesting that predefined categories may not fully capture the diversity and personal specificity of contemporary sexual identities. Future research should continue to refine measures of sexual identity as terminology and patterns of self-identification evolve. Future studies should recruit samples large enough to examine specific identity groups separately, while combining standardized categories with flexible opportunities for self-description and involving diverse adolescents in measure development. Finally, all measures were self-reported and may have been affected by recall and social-desirability biases. Future research could incorporate clinical assessments, service-use data, and qualitative interviews.

Although the substance-use indicators captured frequency of use, they did not assess quantity, motives, context, or resulting impairment. Accordingly, profile labels such as “Cannabis Users” and “Tobacco and E-cigarette Users” describe relative patterns within this sample rather than diagnostic categories or distinctions between experimental, recreational, and problematic use. More detailed assessments may identify additional or more clinically meaningful profiles. Despite these limitations, the use of LPA and EGA provides a strong empirical foundation for future research.

### Policy implications

The results of this study have clear implications for national and international mental health policy, particularly in settings where adolescent services remain underdeveloped, fragmented, or inconsistently accessible. By identifying distinct constellations of psychological symptoms, substance use, and suicidal ideation, and by demonstrating how these profiles intersect with structural, emotional, and identity-related barriers to help-seeking, the study supports a shift from generalized youth mental health policy toward approaches that are responsive to diverse pathways of distress and to the specific social contexts in which adolescents make decisions about whether to seek care.

The profiles observed here illustrate that adolescents are not a homogeneous population. Youth who experience elevated emotional distress and suicidal ideation face internal and interpersonal barriers that may discourage disclosure and make formal services feel inaccessible or unsafe. Adolescents whose difficulties are embedded in patterns of tobacco, e-cigarette, or cannabis use encounter obstacles that are often logistical or financial. These distinctions indicate that policy frameworks should combine universal mental health promotion with targeted pathways responsive to adolescents’ risk profiles and perceived barriers. Youth experiencing co-occurring psychological difficulties and substance use may benefit from integrated or coordinated care rather than referrals between separate services. Particular outreach may be warranted for sexual minority youth in the Tobacco and E-cigarette Users profile, who reported greater identity-related and emotional barriers and lower help-seeking intentions. Low-threshold access through school-based services, confidential walk-in programs, community centers, and telehealth could help reach adolescents who might otherwise avoid traditional clinic-based systems.

The role of sexual orientation in shaping perceived barriers further underscores the need for mental health systems to recognize and address the experiences of sexual minority youth explicitly. Across many countries, formal guidelines for working with sexual minority adolescents remain uneven, and many service systems lack clear standards for creating affirming, culturally competent, and identity-safe environments. The elevated identity-related barriers reported by sexual minority adolescents in this study reflect challenges documented internationally, including concerns about stigma, judgment, misunderstanding, and unwanted disclosure. Embedding identity-affirming principles within professional training, service design, and youth-focused outreach may reduce these barriers and support earlier and safer engagement with care.

The findings also highlight confidentiality as a policy priority. Emotional barriers in this study often centered on fears that seeking help would expose adolescents to parental disapproval or peer scrutiny. Policies should clearly articulate the circumstances under which young people may access care confidentially and provide guidance to clinicians on balancing privacy with safety. School psychological services, community providers, and municipal welfare departments could support early identification, confidential consultation, and active referral while ensuring that adolescents receive clear information about confidentiality and its limits.

Although these recommendations apply broadly across national contexts, they are particularly relevant in countries where sexual minority issues remain socially sensitive or insufficiently addressed in public policy. In Israel, formal guidelines on mental health care for sexual minority youth are still emerging, and open discussion of identity-related needs has historically been limited [38]. The Ministry of Health could lead the development of national standards for confidential, low-threshold, and identity-affirming adolescent mental health services. Israel’s health funds could implement these standards through intake procedures that assess emotional distress, substance use, behavioral difficulties, practical barriers to access, and concerns about identity disclosure or professional misunderstanding. School psychological services, municipal welfare departments, and designated LGBTQ+ social workers could complement these efforts by providing confidential consultation and facilitating active referrals to appropriate health-fund services.

Implementation could begin with a multisector pilot led by the Ministry of Health in collaboration with the health funds, the Ministries of Education and Welfare and Social Affairs, local authorities, and LGBTQ+ youth organizations. The pilot could assess referral completion, waiting times, treatment engagement, perceived barriers, and service acceptability. Israel’s combination of national health insurance, school-based services, and municipal welfare infrastructure provides an appropriate setting for testing such a coordinated approach. If effective, its core components - multidomain assessment, low-threshold access, cross-system coordination, and identity-affirming mainstream care - could subsequently be adapted to other national contexts.

## Conclusions

This study demonstrates that adolescent mental health profiles and help-seeking are associated with distinct patterns of structural, emotional, and identity-related barriers. By illuminating the structural and psychological contours of these experiences, our findings provide a roadmap for more inclusive, nuanced, and effective mental health care strategies for all youth, especially those too often rendered invisible in traditional models of service provision.

**Table 1:** Sociodemographic characteristics of participants (*N* = 1,129)

Characteristic	*N* (%)
**Age** (M, SD)	17.42 (1.03)
**Sex Assigned at Birth**	
Male	264 (23%)
Female	855 (76%)
Prefer not to say	9 (0.8%)
**Gender Identity**	
Man	273 (24%)
Woman	835 (74%)
Non-binary/Other	21 (1.9%)
**Sexual Orientation**	
Heterosexual	791 (70%)
Gay/Lesbian	22 (2.0%)
Mainly heterosexual	49 (4.3%)
Mainly gay/lesbian	8 (0.7%)
Bisexual	87 (7.7%)
Queer	7 (0.6%)
Pansexual	19 (1.7%)
Polysexual	0 (0%)
Asexual	4 (0.4%)
Questioning	63 (5.6%)
Other	77 (6.9%)
**Religion**	
Jewish	1051 (93.09%)
Christian	12 (1.06%)
Muslim	60 (5.31%)
Other	6 (0.53%)
**Religiosity**	
Secular	514 (46%)
Religious	293 (26%)
Traditional	230 (20%)
Ultra-Orthodox	47 (4.2%)
Other	42 (3.7%)
**Parental Marital Status**	
Married	875 (78%)
Divorced	168 (15%)
Single Parent	25 (2.2%)
Widowed	22 (2.0%)
Common-law	16 (1.4%)
Other	22 (2.0%)

**Table 2:** Fit indices for 1 to 6 profile solutions

	1 profile	2 profiles	3 profiles	4 profiles	5 profiles	6 profiles
BIC	32170.21	30376.93	28840.04	28197.93	27741.29	**27653.58**
SABIC	32106.68	30278.47	28706.64	28029.59	27,538	**27415.36**
CAIC	32190.21	30407.93	28882.04	28250.93	27805.29	**27728.58**
AWE	32368.79	30685.91	29259.27	28727.49	**28381.16**	28403.99
BLRT		1870.59	1614.20	719.43	533.96	N/A
BF		8.72^^38^	2.36^^33^	8.77^^13^	8.23^^9^	**80.23**
cmP	0.00	0.00	0.00	0.00	0.01	**0.99**
Entropy	1	0.96	1.00	0.99	1.00	0.89
LL	-16014.81	-15079.51	-14272.41	-13912.7	-13645.71	-13563.2
% smallest n		0.16	0.10	0.07	0.06	0.05

Note. BIC = Bayesian Information Criterion; SABIC = Sample-size adjusted Bayesian Information Criterion; CAIC = Consistent Akaike Information Criterion; AWE = Approximate Weight of Evidence Criterion; BLRT = bootstrapped likelihood ratio test; BF = Bayes Factor; cmP = correct model probability. Bolded values indicate the best fit as reflected by the index. A minimum profile size of 56 participants equals 5.0% or higher

**Table 3:** Comparisons between profiles in participants’ barriers to seeking health care, intentions to seek mental health help, and sexual orientation

Characteristic	Within Normal Ranges *N* = 692^1^	Emotional Struggles and Suicidal Ideation *N* = 251^1^	Tobacco and E-cigarette Users *N* = 77^1^	Cannabis Users *N* = 109^1^	*p*-value^2^	q-value^3^
Intention to seek mental health help	4.77 (1.67)	4.16 (1.96)	4.65 (1.88)	4.35 (1.40)	< 0.001	< 0.001
Identity-Related Barriers	1.71 (0.76)	2.34 (0.94)	2.13 (0.92)	2.18 (0.66)	< 0.001	< 0.001
Accessibility Structural Barriers	2.12 (0.88)	2.53 (0.90)	2.56 (1.04)	2.91 (0.90)	< 0.001	< 0.001
Emotional Internal Barriers	2.28 (0.98)	2.99 (1.04)	2.61 (1.22)	2.78 (0.86)	< 0.001	< 0.001
Sexual Orientation					< 0.001	< 0.001
Heterosexual	567 (82%)	139 (55%)	48 (62%)	79 (72%)		
Sexual minority	125 (18%)	112 (45%)	29 (38%)	30 (28%)		

^1^Mean (SD); n (%)

^2^Fisher’s Exact Test for Count Data with simulated p-value (based on 10000 replicates)

^3^False discovery rate correction for multiple testing

**Table 4:** Robust regressions for predicting barriers and intention to seek mental help by participants’ profile, sexual orientation, and their interaction

	Identity-Related Barriers			Accessibility Structural Barriers		
Predictors	Estimates	CI	p	Estimates	CI	p
(Intercept)	1.69	1.58 – 1.80	**<0.001**	1.9	1.76 – 2.04	**<0.001**
Emotional Struggles vs. WNR	0.57	0.45 – 0.69	**<0.001**	0.37	0.22 – 0.52	**<0.001**
Tobacco vs. WNR	0.46	0.27 – 0.65	**<0.001**	0.45	0.21 – 0.69	**<0.001**
Cannabis vs. WNR	0.5	0.32 – 0.68	**<0.001**	0.77	0.55 – 1.00	**<0.001**
Sexual Orientation	0.37	0.23 – 0.52	**<0.001**	0.12	-0.07 – 0.30	0.208
Sex	0.08	-0.02 – 0.19	0.11	0.29	0.16 – 0.42	**<0.001**
Emotional Struggles vs. WNR x Sexual Orientation	0.25	0.01 – 0.49	**0.038**	-0.04	-0.33 – 0.26	0.817
Tobacco vs. WNR x Sexual Orientation	0.49	0.11 – 0.87	**0.012**	0.08	-0.40 – 0.56	0.756
Cannabis vs. WNR x Sexual Orientation	-0.06	-0.41 – 0.30	0.764	-0.33	-0.79 – 0.12	0.146
Observations	1119			1119		
R^2^ / R^2^ adjusted	0.199 / 0.193			0.110 / 0.104		
	**Emotional Internal Barriers**			**Intentions to Seek Mental Help**		
Predictors	Estimates	CI	p	Estimates	CI	p
(Intercept)	2.11	1.96 – 2.27	**<0.001**	4.84	4.57 – 5.11	**<0.001**
Emotional Struggles vs. WNR	0.7	0.54 – 0.87	**<0.001**	-0.65	-0.94 – -0.35	**<0.001**
Tobacco vs. WNR	0.35	0.09 – 0.62	**0.01**	-0.27	-0.74 – 0.19	0.252
Cannabis vs. WNR	0.49	0.24 – 0.74	**<0.001**	-0.29	-0.73 – 0.15	0.198
Sexual Orientation	0.2	0.02 – 0.39	**0.040**	-0.01	-0.38 – 0.35	0.939
Sex	0.25	0.10 – 0.40	**0.001**	0.01	-0.24 – 0.27	0.915
Emotional Struggles vs. WNR x Sexual Orientation	0.05	-0.28 – 0.38	0.776	-0.05	-0.64 – 0.54	0.879
Tobacco vs. WNR x Sexual Orientation	0.54	0.04 – 1.03	**0.049**	-1.04	-1.97 – -0.10	**0.030**
Cannabis vs. WNR x Sexual Orientation	-0.24	-0.74 – 0.26	0.343	1.05	0.17 – 1.92	**0.019**
Observations	1119			1119		
R^2^ / R^2^ adjusted	0.116 / 0.110			0.036 / 0.029		

**Fig. 4a:**
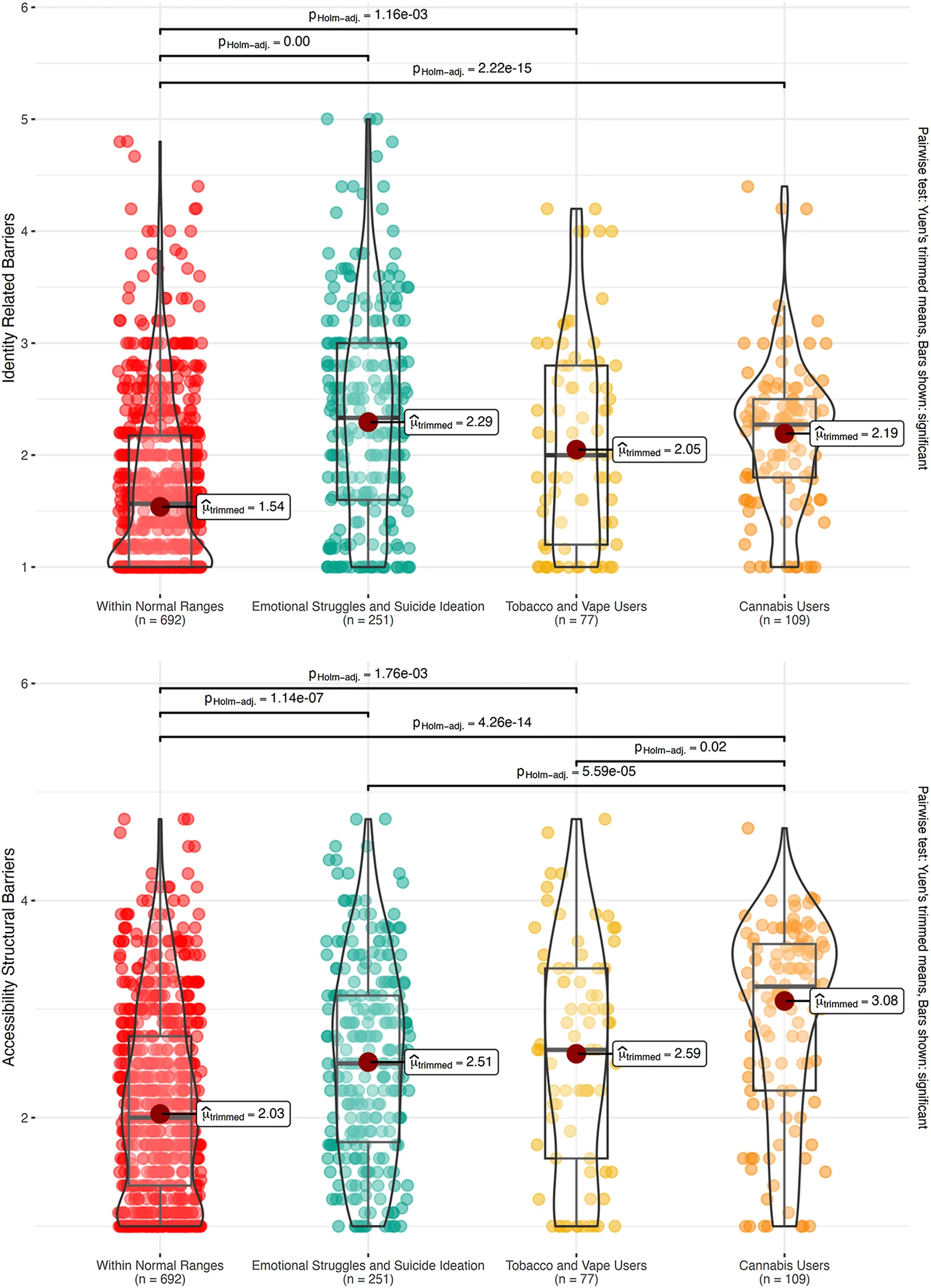
Differences in identity-related barriers and accessibility structural barriers between profiles

**Fig. 4b:**
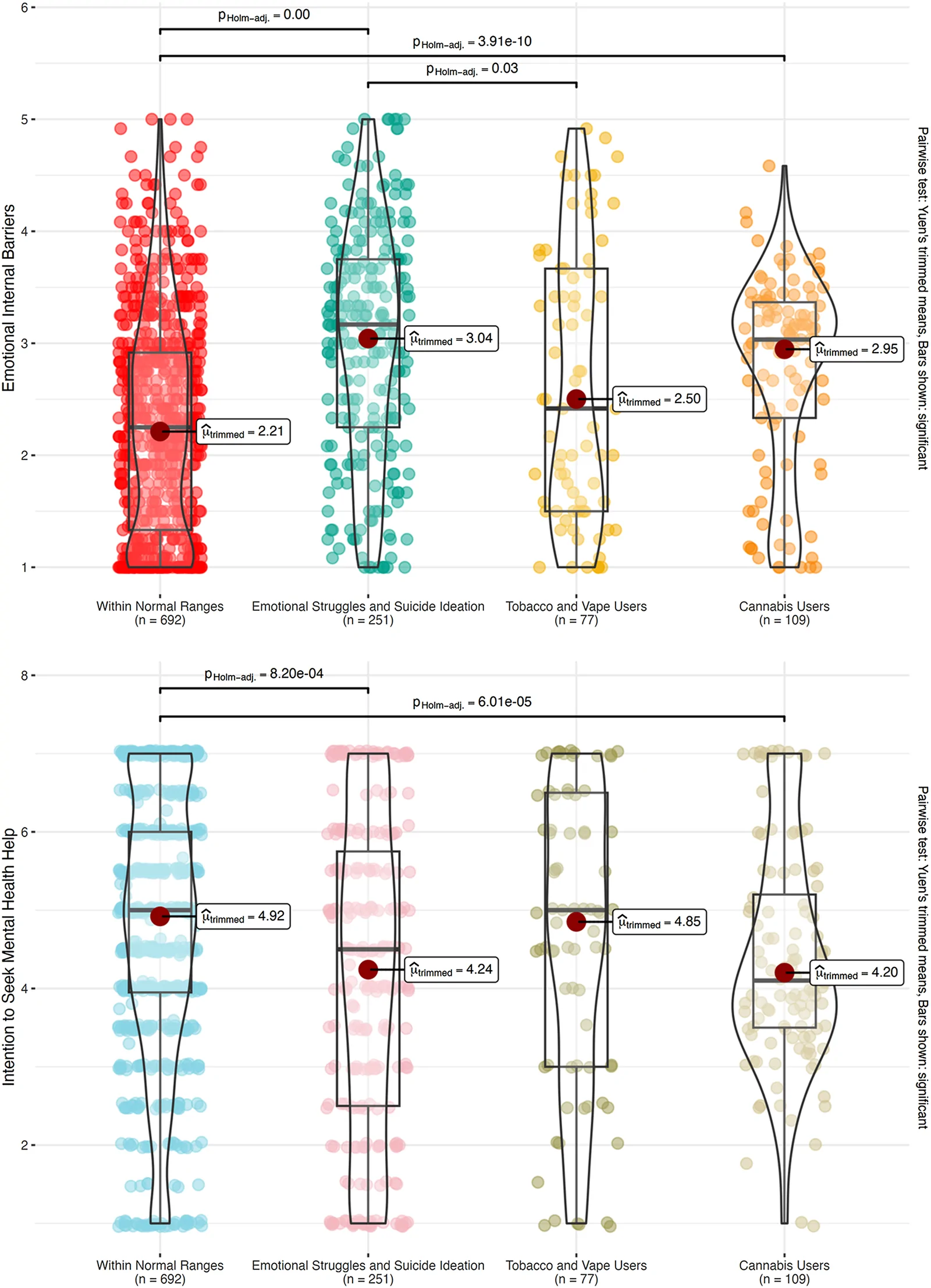
Differences in emotional internal barriers and intentions to seek mental health help between profiles

**Fig. 5a:**
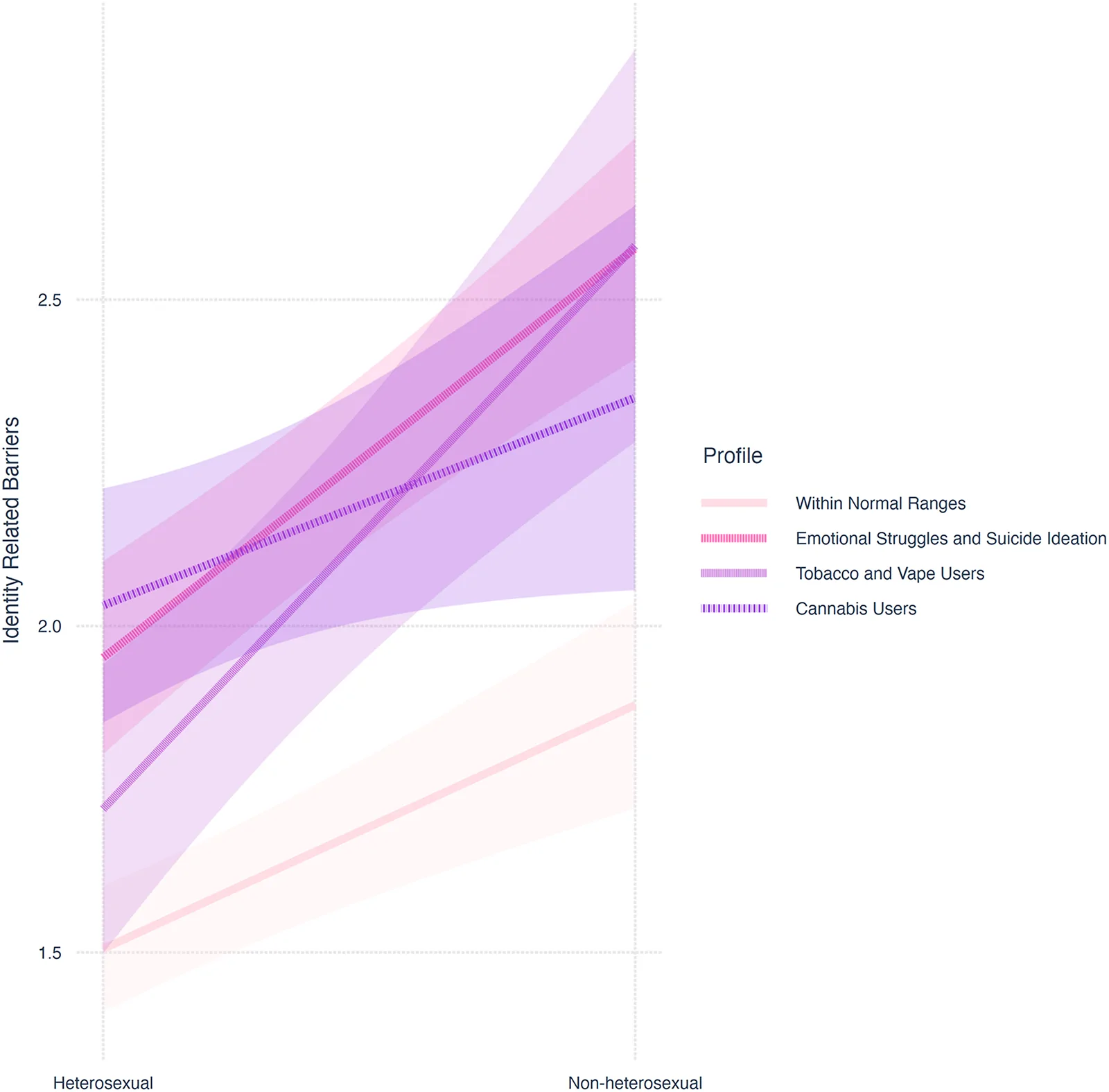
Interaction between profile and sexual orientation in predicting participants’ identity-related barriers

**Fig. 5b:**
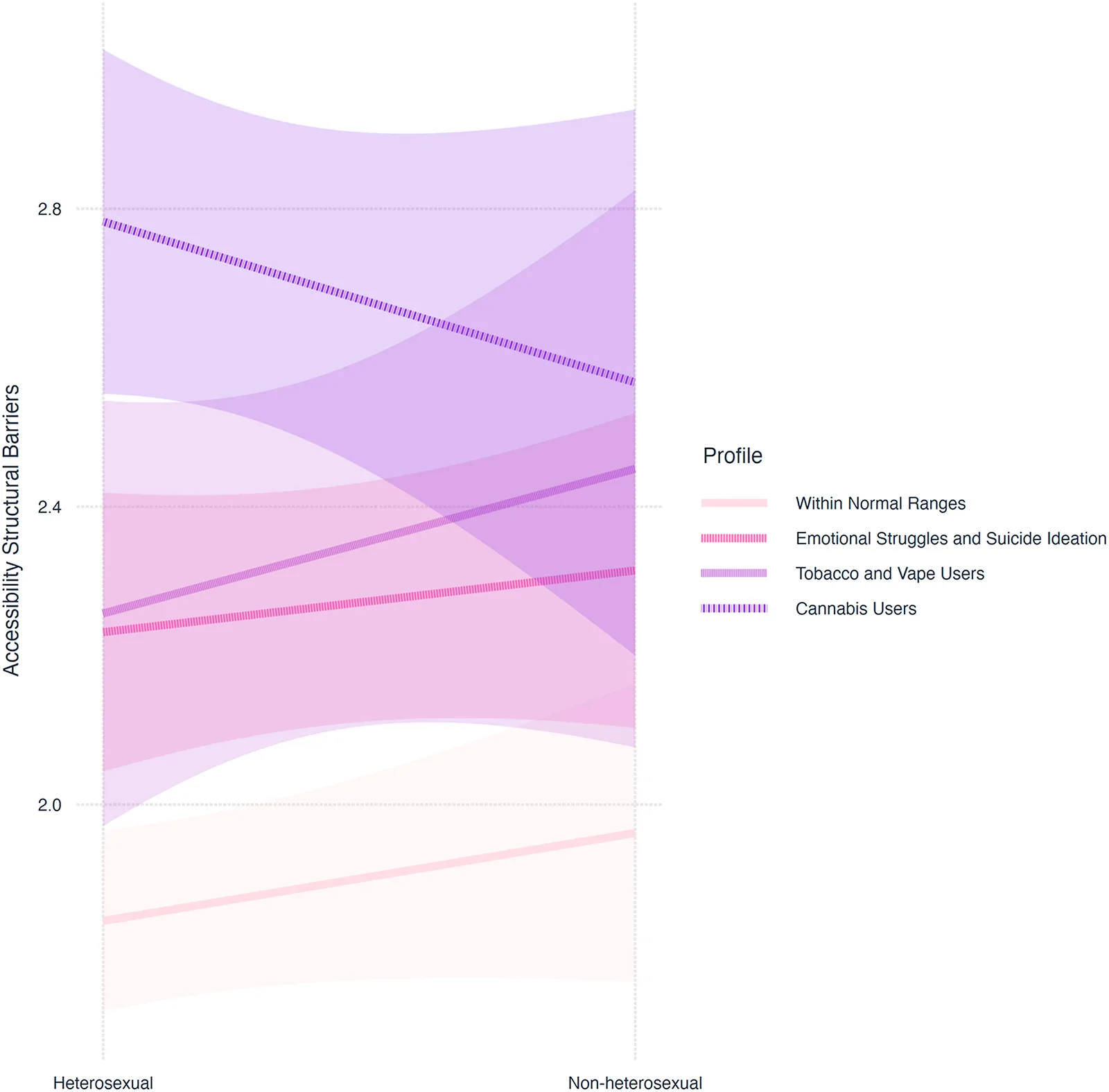
Interaction between profile and sexual orientation in predicting participants’ accessibility structural barriers

**Fig. 5c:**
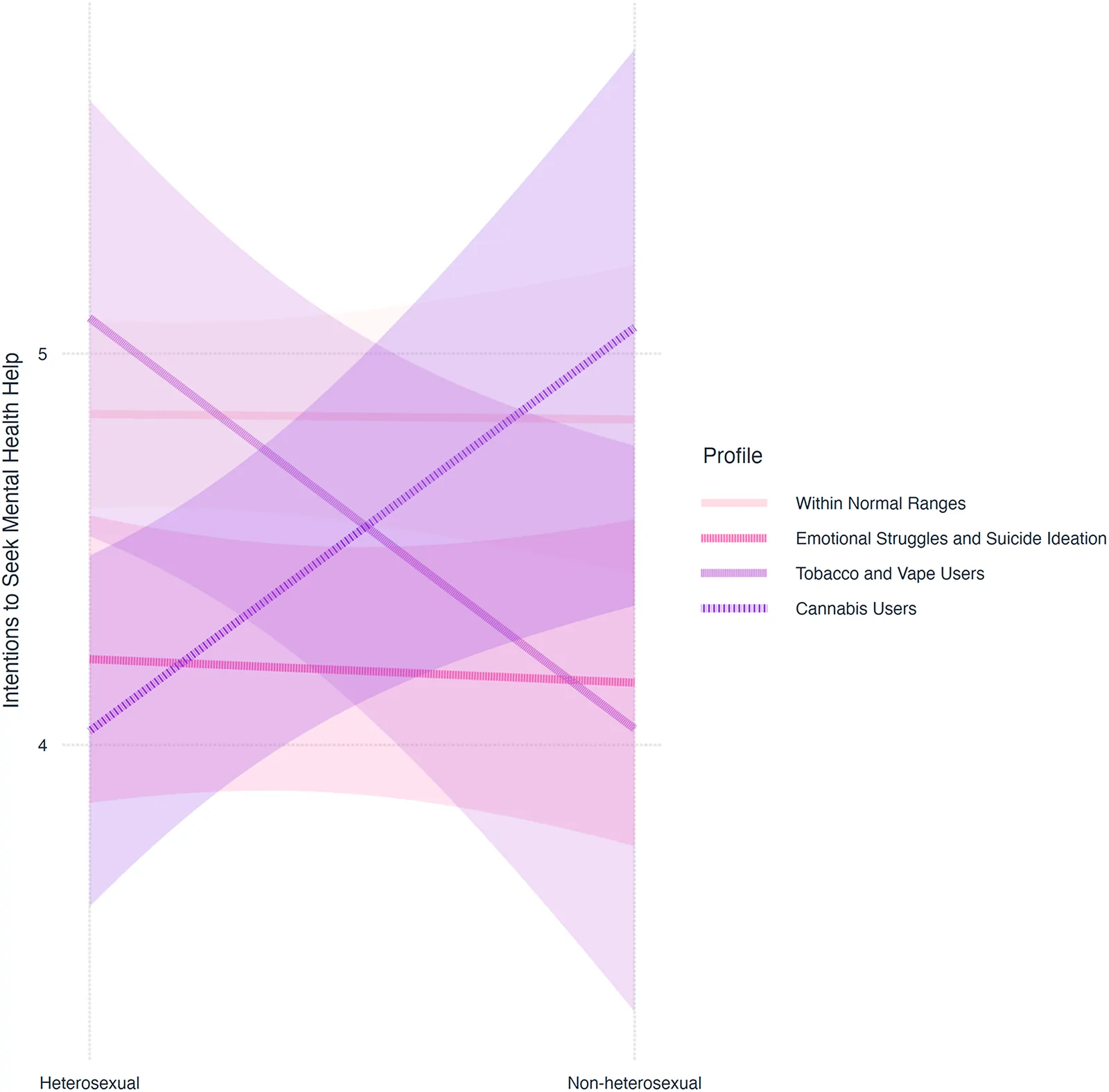
Interaction between profile and sexual orientation in predicting participants’ intentions to seek mental health help

## Electronic Supplementary Material

Below is the link to the electronic supplementary material.

Supplementary Material 1 : Supplementary Table 1 Differences between profiles in the constructs used in the LPA

## Data Availability

No datasets were generated or analysed during the current study.
