# Supplementary material for: Latent profiles of mental health and care barriers among heterosexual and sexual minority adolescents
Source: Isr J Health Policy Res. 2026 Sep 4;15:38. doi: 10.1186/s13584-026-00781-0 (PMC13543487; doi:10.1186/s13584-026-00781-0)
Supplement: Supplementary file 1 [file 13584_2026_781_MOESM1_ESM.docx]

Supplementary Table 1

Differences between profiles in the constructs used in the LPA

| **Characteristic** | **Within Normal Ranges**  N = 692^1^ | **Emotional Struggles and Suicidal Ideation**  N = 251^1^ | **Tobacco and E-cigarette Users**  N = 77^1^ | **Cannabis Users**  N = 109^1^ | **p-value**^2^ | **q-value**^3^ |
| --- | --- | --- | --- | --- | --- | --- |
| Tobacco | 0.16 (0.46) | 0.16 (0.40) | 3.47 (0.68) | 1.56 (1.04) | <0.001 | <0.001 |
| Alcohol | 0.79 (0.88) | 0.87 (0.96) | 1.92 (1.11) | 1.59 (0.77) | <0.001 | <0.001 |
| Cannabis | 0.05 (0.22) | 0.07 (0.25) | 0.25 (0.43) | 2.36 (0.54) | <0.001 | <0.001 |
| E-cigarette | 0.14 (0.44) | 0.33 (0.76) | 1.86 (1.62) | 1.55 (1.02) | <0.001 | <0.001 |
| Emotional Symptoms | 4.23 (2.50) | 6.03 (2.35) | 5.55 (2.87) | 5.18 (2.39) | <0.001 | <0.001 |
| Conduct Problems | 1.83 (1.51) | 2.52 (1.91) | 3.01 (1.81) | 2.66 (1.89) | <0.001 | <0.001 |
| Hyperactivity/Inattention | 3.56 (2.32) | 4.68 (2.38) | 5.18 (2.65) | 5.06 (1.96) | <0.001 | <0.001 |
| Peer Relationship Problems | 2.32 (1.73) | 3.50 (2.00) | 3.35 (2.35) | 3.28 (1.68) | <0.001 | <0.001 |
| Prosocial Behavior | 8.23 (1.67) | 7.95 (1.71) | 8.01 (1.98) | 7.81 (1.92) | 0.037 | 0.040 |
| Suicidal Ideation |  |  |  |  | <0.001 | <0.001 |
| No | 692 (100%) | 0 (0%) | 31 (40%) | 70 (64%) |  |  |
| Yes | 0 (0%) | 251 (100%) | 46 (60%) | 39 (36%) |  |  |
| Sexual Orientation |  |  |  |  | <0.001 | <0.001 |
| Heterosexual | 567 (82%) | 139 (55%) | 48 (62%) | 79 (72%) |  |  |
| Sexual Minority | 125 (18%) | 112 (45%) | 29 (38%) | 30 (28%) |  |  |
| Sex assigned at birth |  |  |  |  | 0.046 | 0.046 |
| Male | 178 (26%) | 43 (17%) | 16 (21%) | 27 (25%) |  |  |
| Female | 510 (74%) | 206 (83%) | 60 (79%) | 79 (75%) |  |  |
| ^1^Mean (SD); n (%) | | | | | | |
| ^2^Kruskal-Wallis rank sum test; Pearson's Chi-squared test | | | | | | |
| ^3^False discovery rate correction for multiple testing | | | | | | |
